# Prevalence and associated factors of attention deficit hyperactivity disorder (ADHD) among Ugandan children; a cross-sectional study

**DOI:** 10.1186/s13034-017-0155-6

**Published:** 2017-04-14

**Authors:** Joan Wamulugwa, Angelina Kakooza, Sabrina Bakeera Kitaka, Joyce Nalugya, Mark Kaddumukasa, Shirley Moore, Martha Sajatovic, Elly Katabira

**Affiliations:** 1 Department of Pediatrics and Child Health, Mulago Hospital and Makerere University School of Medicine, P. O Box 7072, Kampala, Uganda; 2 Department of Psychiatry, Mulago Hospital and Makerere University School of Medicine, P. O Box 7072, Kampala, Uganda; 3 Department of Medicine, College of Health Sciences, Makerere University, P. O Box 7072, Kampala, Uganda; 4 Neurological and Behavioral Outcomes Center, University Hospital Case Medical Center, Case Western Reserve University, 11100 Euclid Ave, Cleveland, OH 44106 USA

**Keywords:** ADHD, DSM IV, Associated factors, Specialized clinic

## Abstract

**Background:**

Attention deficit hyperactivity disorder (ADHD) is a common neuropsychiatric disorder among the children. The burden of ADHD or its associated factors in Uganda are not known. The objective of this study was to determine the prevalence and the associated factors of ADHD among children attending the neurology and psychiatry clinics at Mulago National Referral Hospital.

**Methods:**

Using the disruptive behavior scale (45 items), we investigated the presence of ADHD symptoms among children attending Mulago Hospital. Questionnaires were administered to the primary care-takers of the study participants to gather information on the factors associated with ADHD. All children were subject to a clinical examination. Children presumed to have ADHD, using the aforementioned rating scale were further assessed by a child psychiatrist to confirm the diagnosis and associated co-morbid conditions.

**Results:**

The estimated prevalence of DSM-IV ADHD symptoms was 11%. Children aged less than 10 years were four times likely to have ADHD (OR 4.1, 95% CI 1.7–9.6, p < 0.001). The demographic factors independently associated with ADHD were age less than 10 years, male gender, history of maternal abnormal vaginal discharge during pregnancy, and no formal education or the highest level of education being primary school.

**Conclusion:**

The prevalence of ADHD among children attending the pediatric neurology and psychiatry clinics is high in our settings and is associated with delayed milestones. Early identification and addressing the co-morbid conditions associated with ADHD such as epilepsy, autism spectrum of disorder, conduct disorder, opposition defiant disorder and intellectual disability in our setting is needed.

## Background

Attention deficit and hyperactivity disorder (ADHD) is a common psychiatric manifestation of childhood diseases [1]. ADHD is defined by features of inattention, overactivity, and impulsivity [2]. Male children are affected more than the females [3]. Its prevalence varies between 4 and 12% worldwide [4]. ADHD impacts school performance among school going children, resulting into impulsive actions, restlessness and lack of focus [5]. There is paucity of published data regarding ADHD in sub-Saharan Africa [6] and particularly in Uganda. No studies have been conducted in Uganda to determine the prevalence of ADHD. The prevalence of ADHD varies in the published reports from South Africa, Democratic Republic of Congo, Nigeria or Ethiopia, showing a reported prevalence varying from 5.4 to 8.7% among school children [7–10]. The prevalence of ADHD reported on other continents is variable. In South America, the prevalence of ADHD in children is about 6%, while in the USA it is as high as 16% [1, 11].

The present study was therefore conducted; (a) to determine the prevalence of ADHD among children attending the neurology and psychiatry clinics at Mulago Hospital, and (b) to identify the factors associated with ADHD among children attending Mulago National Referral Hospital.

## Methods

### Design

This was an analytical cross-sectional study of children attending the Mulago National Referral Hospital.

### Setting

The study was done at the pediatric neurology and psychiatry clinics of Mulago National Referral Hospital, the largest hospital in Uganda. It is a teaching hospital of Makerere University College of Health Sciences. The pediatric neurology clinic is an outpatient’s specialized clinic and operates every Thursday from 9:00 a.m. to 3:00 p.m. except on public holidays. The pediatric neurology clinic receives referred patients from all over the country with neurologic complications. About 20 children are seen on each clinic day. The team of health workers during clinic days includes a pediatric neurologist, two senior house officers/residents, a medical officer, two nursing officers and a records clerk. Medications prescribed from the clinic are dispensed at the clinic pharmacy when available. Patients diagnosed with ADHD are sent to the psychiatry clinic to get further assessment from a child psychiatrist and then get the necessary treatment and specific medications.

The child psychiatry clinic at Mulago Hospital is under the Department of Psychiatry and Mental Health. It is also a specialized centre for all mental disorders in the country. It operates as an outpatient’s specialized mental clinic on Tuesdays and Thursdays between 9:00 a.m. and 3:00 p.m. except on public holidays.

On every clinic day, about 10 children are attended to by a team of health workers including a child psychiatrist, a child psychologist, two psychiatric senior house officers/residents, two clinical officers, two nursing officers and two records clerks. Prescribed drugs are dispensed from the psychiatry clinic pharmacy when available.

### Sample size estimation

The sample size was calculated using the formula: $\left\{ {n = \frac{{Z_{\alpha }^{2} (pq)}}{{d^{2} }}} \right\}$ where p = prevalence of ADHD, q = complement of the prevalence, margin of error is error = d, alpha = significance level. Setting the significance at 0.05 and error margin at 5%, we adjusted the sample size requirement for an assumed 30% level of non-response. Based on a previous study in the USA [4] where ADHD prevalence was 12% and N* = 332, we recruited 332 participants.

### Study questionnaire

The disruptive behavior disorders rating scale (DBRS) was completed for each study participant to identify the children who were likely to have ADHD symptoms. The scale consists of 45 items representing symptoms of disruptive behavior disorders including; conduct disorder, oppositional defiant disorder and ADHD. All 45 screening items were scored in the present study. Each symptom is rated on a four-point scale indicating the occurrence and severity or symptoms; 0 (not at all), 1 (just a little) 2 (pretty much) and 3 (very much). The scales were scored using the scoring method described by Pelham [12]. According to the DSM-IV, ADHD is divided into three subtypes that are predominantly inattentive (ADHD-I), predominantly hyperactivity/impulsivity (ADHD-HI) and combined (ADHD-C) [13].

The diagnosis of ADHD was confirmed by the child psychiatrist using the Mini International Neuropsychiatric Interview for Children and Adolescents (MINI Kid) version 6.0, a tool based on DSM IV criteria for diagnosis of psychiatric conditions [14]. The co-morbid conditions coexisting with and factors associated with ADHD were diagnosed using the same tool. The study participants’ care giver/guardians’ were asked if there were any delayed milestones for the children and a history of maternal abnormal vaginal discharge during pregnancy.

### Study subjects

Study subjects were children aged between 4 and 18 years attending the Mulago National Referral Hospital, neurology and psychiatry clinics between 7th August 2014 and 4th June 2015. The inclusion criteria included; children aged between 4 and 18 years attending the neurology and psychiatry outpatient clinics. All children enrolled into the study had to be a companied by adult caregivers who consented for their participation in the study. Children whose caregivers during the clinic visit did not know much about the children’ illness and symptoms were excluded from the study.

### Study procedures

Study participants were approached, screened and consecutively enrolled from the outpatient clinic days until the required sample size was obtained. Identification and screening of the participants were systematically done by the study team in the reception areas. The guardians/parents were approached by the study team for consent to participate in the study. Among study participants age eight or older without severe intellectual disability, assent was sought to participate in the study. The PI or research assistant interviewed the caretakers of study participants or the study participants (those who gave assent) using pretested questionnaire written in English, but administered in the language best understood by the parent/guardian. A structured self-administered questionnaire was used to collect information from the parents of children, as well as older children, who presented to paediatric outpatient clinics during the study period. In a few cases in which the parents were illiterate, the questionnaire was administered by study investigators. Parents were asked to recall symptoms, from a list of criteria for the diagnosis of ADHD, exhibited by their children either at home or at school. We used the DSM-IV-TR diagnostic criteria for ADHD. The responses were recorded in English. The physical examination of the study participant was done by the PI or the research assistant. A medical screening of each study participant, including height, weight, temperature and a review of systems, was conducted by the study pediatricians to identify any existent health problems that required immediate medical treatment. Neurological and mental status examinations were done in detail by study pediatricians. Abnormalities of movement and coordination such as tremors, chorea, athetosis, dystonia, gait and ataxia were also assessed. Children who were identified (using the disruptive rating scale) with symptoms consistent with ADHD were referred to a psychiatrist for further diagnostic assessment and appropriate treatment including long term management. All children with ADHD were confirmed by a child psychiatrist.

### Statistical analysis

All questionnaires were cross-checked for completeness, sorted, coded and entered into the computer using Epidata version 2.1 packages. The raw data was securely stored to maintain confidentiality. Data was analyzed with the help of a statistician using Stata version 12.0 software (StataCorp. 2011. Stata Statistical Software: Release 12. College Station, TX: StataCorp LP).

## Results

### General description

A total of 520 children were screened for the study. Of these, 188 participants were excluded from the study as follows: 173 participants were not in the age bracket for the inclusion criteria, 10 participants had missing data, and 5 participants lacked the caretaker’s consent to participate in the study. Therefore, 332 participants were recruited and enrolled to participate in the study. Among these study participants with ADHD, 56% were from psychiatry clinic while 44% were from neurology clinic. Two children were receiving phenobarbitone while 18 were receiving benzodiazepines for their epilepsy.

### Estimated prevalence of ADHD

The prevalence of ADHD in this sample is 11.7% (39/332), with a prevalence of 12.1% amongst participants who attended neurology clinic and a prevalence of 11.5% amongst participants who attended psychiatry clinic. A prevalence of 14.9% was noted among male study participants compared to a prevalence of 7.6% among female study participants.

### Associations between baseline characteristics and ADHD among the study participants

Eighty-two percent of the participants with ADHD were less than 10 years old and it was noted that those below the age of 10 years old were four times more likely to have ADHD (OR 4.1, 95% CI 1.7–9.6, p < 0.001). Similarly, 82% participants who came in with their mothers as next of kin were two times more likely to have ADHD (OR 2.8, 95% CI 1.2–6.7, p = 0.011). Seventy-one percent of the study participants with ADHD had a history of delayed milestones as identified by the study pediatricians. Among these study participants, delayed milestones was significantly associated with ADHD (p = 0.001) (Table 1).

**Table 1:** Unadjusted analysis for baseline characteristics and ADHD among children attending the paediatric neurology and psychiatry Clinics of Mulago Hospital

Baseline characteristics and clinic participant distribution	ADHD N (%) N = 39	No ADHD N (%) N = 293	Unadjusted OR (95% CI)	p value
Clinic				
Neurology^†^	17 (43.5)	123 (41.9)	1.00	
Psychiatry	22 (56.4)	170 (58.0)	0.94 (0.45–1.96)	0.848
Age categories in years				
>10^†^	7 (17.9)	139 (47.4)	1.00	
≤10	32(82.0)	154 (52.5)	4.1 (1.7–9.6)	*<0.001*
Gender				
Female^†^	11 (28.2)	133 (45.3)	1.00	
Male	28 (71.7)	160 (54.6)	2.1 (1.0–4.4)	*0.042*
Relationship with next of kin				
Other^†^	7 (17.9)	111 (38.6)	1.00	
Mother	32 (82.0)	176 (61.3)	2.8 (1.2–6.7)	0.011
Child has delayed milestones				
No^†^	11 (28.2)	169 (57.6)	1.00	
Yes	28 (71.7)	124 (42.3)	3.4 (1.6–7.2)	*0.001*
Is child above 6 years attending school				
No^†^	19 (48.7)	93 (31.7)	1.00	
Yes	20 (51.2)	200 (68.2)	0.5 (0.2–1.0)	0.035

^†^Fisher’s exact p value and Reference category

Significant p values less than 0.05 are in italics

### Adjusted analysis for factors associated with ADHD

At adjusted analysis, the factors that were significantly associated with ADHD included: age less than 10 years of the participant (p 0.003), male gender of the participant (p 0.017), and maternal abnormal vaginal discharge during pregnancy (p 0.004). The study participant’s medical history of epilepsy (p 0.015) was associated with ADHD. A participant who is younger than age 10 was four times more likely to have ADHD (OR 4.32, 95% CI 1.65–11.33). A male participant was three times more likely to have ADHD than female participants (OR 2.87, 95% CI 1.21–6. 81). Children born to a mother with history of abnormal vaginal discharge during pregnancy were four times more likely to have ADHD (OR 3.89, 95% CI 1.54–9.79). A participant with a caretaker who had no formal education or had primary education as the highest level of education was three times more likely to have ADHD (OR 3.16, 95% CI 1.35–7.37; p value 0.030) (Table 2).

**Table 2:** Unadjusted and adjusted analysis for factors associated with ADHD

	Unadjusted OR (95% CI)	p value	Adjusted OR (95% CI)	p value
Age (years)				
>10^a^	1.00		1.00	
≤10	4.13 (1.76–9.65)	0.001	4.32 (1.65–11.33)	*0.003*
Gender				
Female^a^	1.00		1.00	
Male	2.12 (1.02–4.41)	0.045	2.87 (1.21–6.81)	*0.017*
Relationship with next of kin				
Other^a^	1.00		1.00	
Mother	3.04 (1.30–7.11)	0.010	6.96 (1.65–29.30)	0.008
Abnormal vaginal discharge during pregnancy				
No^a^	1.00		1.00	
Don’t know	1.11 (0.46–2.69)	0.813	3.60 (0.87–14.92)	0.078
Yes	4.54 (2.04–10.09)	<0.001	3.89 (1.54–9.79)	*0.004*
Caretaker level of education				
Post primary^a^	1.00		1.00	
Don’t know	3.65 (0.90–14.83)	0.071	8.04 (1.22–52.91)	0.030
None/primary	2.01 (1.00–4.04)	0.051	3.16 (1.35–7.37)	*0.008*
Epilepsy medical history				
No^a^	1.00		1.00	
Yes	0.48 (0.24–0.95)	0.034	0.36 (0.16–0.82)	0.015
Known family history of ADHD				
No^a^	1.00		1.00	
Don’t know	2.55 (1.05–6.19)	0.038	3.13 (1.04–9.45)	0.043
Yes	3.14 (1.28–7.74)	0.013	1.91 (0.67–5.46)	0.229

^a^Reference category

Significant p values less than 0.05 are in italics

### Co-morbidities associated with ADHD

Children with ADHD were further screened for other comorbidities. The frequency of these co-morbidities associated with ADHD were epilepsy (25.71%), autism spectrum disorders (14.29%), conduct disorder (8.57%) and intellectual disability (8.57%).

**Fig. 1:**
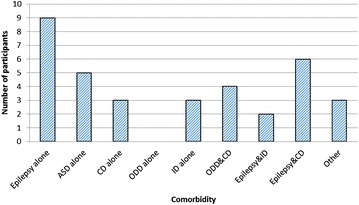
Shows the frequency of disease comorbidities among the study participants with epilepsy and autism spectrum of disorders the commonest comorbidities

Some participants with ADHD had more than one co-morbid condition. The most common combination was epilepsy and conducts disorder (17.14%), and oppositional defiant disorder (ODD) and conduct disorder (CD) (11.43%), and epilepsy and intellectual disability (ID) (5.71%) (Fig. 1). There was no participant with oppositional defiant disorder while 7.7% (3/39) had conduct disorder alone. Ten point three percent (4/39) had both ODD and CD combined.

## Discussion

This study set out to determine the prevalence and associated factors of attention deficit hyperactivity disorder among children attending the pediatric neurology and psychiatry clinics at Mulago National Referral Hospital.

### The prevalence of ADHD

The prevalence of ADHD in our sample was 11.7% which is higher than the prevalence reported in prior African samples. A prior study found the prevalence of ADHD to be 6% among school children ages 7–9 years, from ten randomly selected schools in Kinshasa, Congo [15]. Adewuya et al. found a prevalence of 8.7% among primary school children ages 7–12 years in Nigeria [9]. The prevalence of ADHD in the clinic sample was higher than the prevalence found in the previously reported school samples. This difference is likely attributable to the different types of sample settings, i.e. a school versus a clinic. Children attending the neurology and psychiatry clinics from this sample are typically referred from other hospitals for specialized care and are often referred because an underlying neurologic or psychiatric condition is already suspected. The prevalence in this sample might not be a true reflection of the overall burden of illness in the country. Of note, some studies have indicated that culture and geographical location may have little or no influence on the prevalence of ADHD [1, 9]. While the prevalence of ADHD in Africa was previously reported between 5.4 and 8.7% [8, 9, 16, 17] in school going children samples, and 1.5% among the general community [18]. Our finding of the prevalence of ADHD at 11.7% in a clinic sample is higher probably because the study participants in this study were obtained from a clinical setting; which is a highly specialized population. Other experts have argued that the variability of ADHD/HD prevalence estimates may be best explained by the use of different case definitions and that no variability of the actual prevalence across geographical sites should be found when case definitions are the same [19–21].

### Factors associated with ADHD

In this study, the male participants were three times more likely to have ADHD than the female participants. In this study the prevalence of ADHD was 8.4% in males and 3.3% in female participants aged 4–18 years. However, the observation in this study has been previously reported in other studies. Peter Szatmari et al. [22] reported a prevalence of 9% among boys and that of 3.3% among girls, in an Ontario child health survey. Steven P Cuffe in a national health survey of a household population in the United Stated of America observed a prevalence of 6.8% among males and that of 2.5% among female children [23]. Although this study did not categorize the subtypes of the ADHD among the study participants, this observation of a higher prevalence of ADHD in male children can be explained by the fact that female children have the inattentive type of the ADHD; as observed by Biederman et al. [24]. Although, our study did not investigate any specific etiological factors associated with ADHD, these findings suggest that this may be worthwhile for future research to explore the possible mechanisms.

This study also observed that age less than 10 years was significantly associated with ADHD. Children less than 10 years were four times more likely to have ADHD. This observation might be attributed to having more children below 10 years (56%) attending the neurology and psychiatry clinics. Reported studies on ADHD among children have been done on different age groups. Biederman et al observed a decline in ADHD symptoms with increasing age among different age groups of children with ADHD over a period of 4 years [25]. This possibly explains why more children with age less than 10 years had symptoms for ADHD compared to those with age of more than 10 years.

This study also found that abnormal vaginal discharge during first trimester of maternal pregnancy was significantly associated with ADHD. This finding could be explained by a possibility of the fetus being exposed to perinatal infections like TORCHES (Toxoplasmosis, Rubella, and Cytomegalovirus, Herpes simplex, Human immunodeficiency virus and syphilis). In this study, systematic screening for these maternal viral infections in the first trimester of pregnancy was not done. Mann Joshua et al. observed that school aged children born to mothers with a history of genitourinary infections were more likely to have ADHD. The study also observed that these mothers reported symptoms of abnormal vaginal discharge and urinary tract infections during their pregnancies [26]. This could possibly explain the relationship between abnormal vaginal discharge and ADHD in this study.

This study found that a child whose primary caretaker had either no education or had primary education as their highest level of education was significantly associated with ADHD. This could be explained by the possibility that the caretaker of this child may have had undiagnosed ADHD in childhood which negatively impacted on their educational attainment. Biederman et al. in an overview of ADHD noted that 5–66% of children with ADHD persist with the disorder to adulthood and that parents of children with ADHD were likely to have ADHD [3]. Sixty-four percent of the study participant had mothers as their primary caretakers. It is possible that some of these mothers had undiagnosed ADHD which persisted into adulthood.

This study also found that epilepsy was significantly protective against ADHD. This is a surprising finding because scientifically, epilepsy is thought to possibly increase the likelihood of having ADHD. Koneski et al. [27] in a review article identifies possible common pathophysiological mechanisms between epilepsy and ADHD, which may help further understand the high prevalence of ADHD among epilepsy patients. The finding of epilepsy being protective against ADHD in this study could be explained by having epilepsy as the most common condition among study participants (71%) and yet a smaller proportion of the participants had ADHD (11.7%) compared to the bigger proportion of the participants (88.3%) who did not have ADHD. It might also be due to the fact that some of the AEDs, such as phenobarbital and benzodiazepines might have a negative effect on attention. The co-morbid conditions observed among participants with ADHD in this study were; epilepsy, autism spectrum of disorders, conducts disorders and intellectual disabilities.

Larson et al. in a meta-analysis to determine patterns of comorbidity among children aged 6–17 years in the United States of America observed that children with ADHD had at least one co-morbid condition like learning disability, conduct disorder and anxiety disorder [28]. Spencer et al. [29] has reported that opposition defiant disorder and conduct disorder co-occurred in 30–50% of children with ADHD. Adewuya et al. in a study among Nigerian school children of aged 7–17 years found that opposition defiant disorder, conduct disorder and anxiety disorder were co-morbid in those with ADHD [9]. The co-morbid conditions differ in these studies as we may speculate that clinicians may be reporting only dominant comorbidities among this population.

This study had the following limitations: recall bias for mothers, especially regarding vaginal discharge and delayed milestones. It is especially difficult to establish an ADHD diagnosis in children younger than age 4 or 5 years, because their characteristic behavior is much more variable than that of older children. However, in this study only a few children were less than 5 years. We did not describe comorbidities like tic or anxiety disorders. The associated factors that were found to be significant in this study would require more exploration so that more information to be obtained from caretakers of study participants to ascertain their true associations, given that this was cross-sectional survey and it may not clearly explain these associations from our results.

Despite these limitations, this study is important because it is the first study in Uganda that estimated the prevalence and the associated factors of ADHD among children. Also, study participants who were presumed to be having ADHD using the DBRS were re-assessed by the child psychiatrist to confirm the diagnosis based of ADHD and its co-morbidities.

## Conclusion

The prevalence of ADHD in our setting was similar to that in other parts of the world though higher than the prevalence previously reported in other African study samples. ADHD was associated with delayed milestones. There is need for additional studies regarding ADHD in this region. Early detection and instituting proper care is important to reduce the impact of ADHD on education of these young children. Untreated ADHD also poses a tremendous amount of psychological and social burden to the individual and the community.

## References

[1] Polanczyk G, de Lima MS, Horta BL, Biederman J, Rohde LA (2007). The worldwide prevalence of ADHD: a systematic review and metaregression analysis. Am J Psychiatr.

[2] American Academy of Pediatrics (2000). Clinical practice guideline: diagnosis and evaluation of the child with attention-deficit/hyperactivity disorder. Pediatrics.

[3] Biederman J (2005). Attention-deficit/hyperactivity disorder: a selective overview. Biol Psychiatr.

[4] Brown RTFW, Perrin JM, Stein MT, Amler RW, Feldman HM, Pierce K, Wolraich ML (2001). Prevalence and assessment of attention-deficit/hyperactivity disorder in primary care settings. Pediatrics.

[5] Merrell CTP (2001). Inattention, hyperactivity and impulsiveness: their impact on academic achievement and progress. Br J Educ Psychol.

[6] Bakare M (2012). Attention deficit hyperactivity symptoms and disorder (ADHD) among African children: a review of epidemiology and co-morbidities. Afr J Psychiatr.

[7] Chinawa JM, Odetunde OI, Obu HA, Chinawa AT, Bakare MO, Ujunwa FA (2014). Attention deficit hyperactivity disorder: a neglected issue in the developing world. Behav Neurol.

[8] Kashala E, Tylleskar T, Elgen I, Kayembe KT, Sommerfelt K (2005). Attention deficit and hyperactivity disorder among school children in Kinshasa, Democratic Republic of Congo. Afr Health Sci.

[9] Adewuya AO, Famuyiwa OO (2007). Attention deficit hyperactivity disorder among Nigerian primary school children: prevalence and co-morbid conditions. Eur Child Adolesc Psychiatr.

[10] Ashenafi Y, Kebede D, Desta M, Alem A (2000). Socio-demographic correlates of mental and behavioural disorders of children in southern Ethiopia. East Afr Med J.

[11] Rohde LA, Biederman J, Busnello EA, Zimmermann H, Schmitz M, Martins S, Tramontina S (1999). ADHD in a school sample of Brazilian adolescents: a study of prevalence, co-morbid conditions, and impairments. J Am Acad Child Adolesc Psychiatr.

[12] Pelham WE, Gnagy EM, Greenslade KE, Milich R (1992). Teacher ratings of DSM-III-R symptoms for the disruptive behavior disorders. J Am Acad Child Adolesc Psychiatr.

[13] Silva RR, Alpert M, Pouget E, Silva V, Trosper S, Reyes K, Dummit S (2005). A rating scale for disruptive behavior disorders, based on the DSM-IV item pool. Psychiatr Q.

[14] Sheehan DVSK, Shytle RD, Janavs J, Bannon Y, Rogers JE, Milo KM, Stock SL, Wilkinson B (2010). Reliability and validity of the Mini International Neuropsychiatric Interview for Children and Adolescents (MINI-KID). J Clin Psychiatr.

[15] Kashala E, Tylleskar T, Elgen I, Kayembe K, Sommerfelt K (2007). Attention deficit and hyperactivity disorder among school children in Kinshasa, Democratic Republic of Congo. Afr Health Sci.

[16] Meyer A (1998). Attention deficit/hyperactivity disorder among North Sotho speaking primary school children in South Africa: prevalence and sex ratios. J Psychol Afr.

[17] Meyer AED, Sundet JM, Tshifularo JG, Sagvolden T (2004). Cross cultural similarities in ADHD-like behavior amongst South African primary school children. S Afr J Psychol.

[18] Ashenafi YKD, Desta M, Alem A (2001). Prevalence of mental and behavioural disorders in Ethiopian children. East Afr Med J.

[19] Swanson JMSJ, Taylor E, Sonuga-Barke EJ, Jensen PS, Cantwell DP (1998). Attention-deficit hyperactivity disorder and hyperkinetic disorder. Lancet.

[20] Bird HR, Jenkins PCJ, Kingston NJ (2002). The diagnostic classification, epidemiology and cross-cultural validity of ADHD. Attention deficit hyperactivity disorder state of the science: best practices.

[21] LA Rohde SC, Polanczyk G, Schmitz M, Martins S, Tramontina S (2005). Attention-deficit/hyperactivity disorder in a diverse culture: do research and clinical findings support the notion of a cultural construct for the disorder?. Biol Psychiatr.

[22] Szatmari P, Offord DR, Boyle MH (1989). Ontario Child Health Study: prevalence of attention deficit disorder with hyperactivity. J Child Psychol Psychiatr.

[23] Cuffe SP, Moore CG, McKeown RE (2005). Prevalence and correlates of ADHD symptoms in the national health interview survey. J Atten Disord.

[24] Biederman JFS, Mick E, Williamson S, Wilens TE, Spencer TJ, Weber W, Jetton J, Kraus I, Pert J, Zallen B (1999). Clinical correlates of ADHD in females: findings from a large group of girls ascertained from pediatric and psychiatric referral sources. J Am Acad Child Adolesc Psychiatr.

[25] Biederman J, Mick E, Faraone SV (2000). Age-dependent decline of symptoms of attention deficit hyperactivity disorder: impact of remission definition and symptom type. Am J Psychiatr.

[26] Mann JR, McDermott S (2010). Are maternal genitourinary infection and pre-eclampsia associated with adhd in school-aged children?. Atten Disord.

[27] Koneski JA, Casella EB (2010). Attention deficit and hyperactivity disorder in people with epilepsy: diagnosis and implications to the treatment. Arq Neuropsiquiatr.

[28] Larson K, Russ SA, Kahn RS, Halfon N (2011). Patterns of comorbidity, functioning, and service use for US children with ADHD, 2007. Pediatrics.

[29] Spencer TJ, Biederman J, Mick E (2007). Attention-deficit/hyperactivity disorder: diagnosis, lifespan, comorbidities, and neurobiology. Ambul Pediatr.

